# An open-label, positron emission tomography study of the striatal D_2_/D_3_ receptor occupancy and pharmacokinetics of single-dose oral brexpiprazole in healthy participants

**DOI:** 10.1007/s00228-020-03021-9

**Published:** 2020-11-16

**Authors:** Dean F. Wong, Arash Raoufinia, Patricia Bricmont, James R. Brašić, Robert D. McQuade, Robert A. Forbes, Tetsuro Kikuchi, Hiroto Kuwabara

**Affiliations:** 1 Lab of CNS Neuropsychopharmacology And Multimodal Imaging (CNAMI), Mallinckrodt Institute of Radiology, Washington University in St. Louis, 4525 Scott Avenue Suite 3114, St. Louis, MO 63110 USA; 2 Otsuka Pharmaceutical Development & Commercialization Inc, Princeton, NJ USA; 3 Section of High Resolution Brain PET, Division of Nuclear Medicine and Molecular Imaging, The Russell H. Morgan Department of Radiology and Radiological Science, Johns Hopkins University School of Medicine, Baltimore, MD USA; 4 Otsuka Pharmaceutical Co., Ltd, Tokushima, Japan

**Keywords:** Antipsychotic agents, Brexpiprazole, Dopamine receptors, Dose determination, Positron-emission tomography, Raclopride, Receptor occupancy, Target engagement

## Abstract

**Purpose:**

The aim of this Phase 1, open-label, positron emission tomography (PET) study was to determine the degree of striatal D_2_/D_3_ receptor occupancy induced by the serotonin–dopamine activity modulator, brexpiprazole, at different single dose levels in the range 0.25–6 mg.

**Methods:**

Occupancy was measured at 4 and 23.5 h post-dose using the D_2_/D_3_ receptor antagonist [^11^C]raclopride. The pharmacokinetics, safety and tolerability of brexpiprazole were assessed in parallel.

**Results:**

Fifteen healthy participants were enrolled (mean age 33.9 years; 93.3% male). Mean D_2_/D_3_ receptor occupancy in the putamen and caudate nucleus increased with brexpiprazole dose, leveled out at 77–88% with brexpiprazole 5 mg and 6 mg at 4 h post-dose, and remained at a similar level at 23.5 h post-dose (74–83%). Estimates of maximum obtainable receptor occupancy (O_max_) were 89.2% for the putamen and 95.4% for the caudate nucleus; plasma concentrations predicted to provide 50% of O_max_ (EC_50_) were 8.13 ng/mL and 7.75 ng/mL, respectively. Brexpiprazole area under the concentration–time curve (AUC_∞_) and maximum plasma concentration (C_max_) increased approximately proportional to dose. No notable subjective or objective adverse effects were observed in this cohort.

**Conclusion:**

By extrapolating the observed single-dose D_2_/D_3_ receptor occupancy data in healthy participants, multiple doses of brexpiprazole 2 mg/day and above are expected to result in an efficacious brexpiprazole concentration, consistent with clinically active doses in schizophrenia and major depressive disorder.

**Trial registration:**

ClinicalTrials.gov NCT00805454 December 9, 2008.

**Electronic supplementary material:**

The online version of this article (10.1007/s00228-020-03021-9) contains supplementary material, which is available to authorized users.

## Introduction

Brexpiprazole (7-{4-[4-(1-benzothiophen-4-yl)piperazin-1-yl]butoxy}quinolin-2(1*H*)-one; chemical structure shown in Fig. S1 in the Online Resource) is a serotonin–dopamine activity modulator that acts as a partial agonist at serotonin 5-HT_1A_ (K_i_ = 0.12 nM) and dopamine D_2_ (K_i_ = 0.30 nM) receptors, and as an antagonist at serotonin 5-HT_2A_ (K_i_ = 0.47 nM) and noradrenaline α_1B_/α_2C_ (K_i_ = 0.17/0.59 nM) receptors, all with subnanomolar affinity [1]. Brexpiprazole also has nanomolar affinity (K_i_ < 5 nM) for dopamine D_3_ receptors (as a partial agonist; K_i_ = 1.1 nM), serotonin 5-HT_2B_/5-HT_7_ receptors (as an antagonist; K_i_ = 1.9/3.7 nM), and noradrenaline α_1A_/α_1D_ receptors (K_i_ = 3.8/2.6 nM) [1]. In comparison to brexpiprazole, the dopamine receptor partial agonist, aripiprazole, has similar affinity at D_2_ (K_i_ = 0.87 nM) and D_3_ (K_i_ = 1.6 nM) receptors, but ten times lower affinity for 5-HT_1A_ (K_i_ = 1.3 nM) and 5-HT_2A_ (K_i_ = 4.7 nM) receptors [1].

The efficacy and safety of brexpiprazole as monotherapy in schizophrenia and as adjunctive therapy to antidepressant treatment in major depressive disorder (MDD) have been demonstrated in several randomized, double-blind, placebo-controlled studies [2–8]. Brexpiprazole is approved in various countries and regions for the treatment of schizophrenia and the adjunctive treatment of MDD in adults.

Positron emission tomography (PET) studies of typical and atypical antipsychotics that act as D_2_ receptor antagonists have shown that the degree of striatal D_2_/D_3_ receptor occupancy provides evidence of target engagement and the window for clinical improvement in schizophrenia, and also the minimal threshold for predicting side effects such as akathisia, hyperprolactinemia, and extrapyramidal symptoms [9–11]. According to PET studies of multiple different antipsychotic drugs at conventional doses, the optimal striatal D_2_/D_3_ receptor occupancy by an antagonist to achieve a clinical effect in schizophrenia with a low incidence of side effects is in the region of 65–80% [9, 11, 12]. Aripiprazole exhibits a higher D_2_/D_3_ receptor occupancy at clinically relevant doses (closer to 90% or 95%), without increasing the risk of extrapyramidal symptoms, because it acts as a partial agonist at these dopamine receptors [12, 13]; in other words, even though the occupancy with aripiprazole is greater than that with antagonists, the functional antagonism may be less due to its partial agonist activity [14].

The aim of this open-label PET study – the first human D_2_/D_3_ receptor target engagement study to be conducted with brexpiprazole – was to determine the degree of striatal D_2_/D_3_ receptor occupancy induced by brexpiprazole at different single dose levels, using the D_2_/D_3_ receptor antagonist [^11^C]raclopride. Pharmacokinetics, safety, and tolerability were also assessed.

## Materials and methods

This study (ClinicalTrials.gov identifier: NCT00805454) was conducted in accordance with the International Conference on Harmonisation Good Clinical Practice Guideline [15] and the Declaration of Helsinki [16]. The study protocol was approved by the Johns Hopkins School of Medicine Institutional Review Board, and written informed consent was obtained from all individual participants included in the study. The study started on November 25, 2008, and was completed on July 17, 2009.

### Participants

Participants were enrolled at two centers in the United States: Johns Hopkins Hospital (Baltimore) and a local contract research organization (SNBL Clinical Pharmacology Center, Baltimore). The key inclusion criteria were that participants (male or female of non-child-bearing potential) must be healthy, aged 18–45 years, and have a body mass index (BMI) of 19–32 kg/m^2^. Key exclusion criteria were a positive alcohol or drug screen, having smoked within 2 months, having used any medication or vitamin supplement in the 14 days prior to dosing (30 days for antibiotics), and any previous exposure to antipsychotics. Participants were also excluded if they had a history of serious medical, neurological, and mental disorders. Participants were screened for the presence of abnormalities on a comprehensive metabolic panel, complete blood counts, liver and renal function tests, an electrocardiogram, urinalysis, and a structural magnetic resonance imaging (MRI) scan of the brain (described below). As an exploratory study, no formal sample size calculation was performed; up to 25 participants were planned for enrollment.

### Study design

This was a Phase 1, open-label, single-dose, PET and pharmacokinetic study (study design shown in Fig. S2 in the Online Resource). The primary objective was to determine the degree of striatal D_2_/D_3_ receptor occupancy induced by brexpiprazole at different single dose levels. Secondary objectives were to determine the pharmacokinetics of brexpiprazole and its main metabolite following a single oral dose, and to determine the safety and tolerability of brexpiprazole following a single oral dose. Using observed data from various single-dose administrations, predictions of D_2_/D_3_ receptor occupancy at expected multiple-dose plasma concentrations were also made.

Participants were screened from day − 29 to day − 2, and eligible participants were checked in to the clinic on day − 1, where they remained until day 7 (except when transported to the PET center for post-dose PET scans, described below).

Participants received a single dose of brexpiprazole, in the range of 0.25–6 mg, on day 1. The first two participants received 0.5 mg; the dose for each subsequent two participants could be increased, repeated, or decreased based on results from the previous two participants. Brexpiprazole was taken with water following a minimum 2 h fast. Participants took no medications other than brexpiprazole during the course of the study. Participants were prohibited to consume grapefruit, grapefruit juice, Seville oranges, or Seville orange juice in the 72 h prior to dosing. In addition, participants were not allowed to consume alcohol, or food and beverages containing methylxanthines (caffeinated coffee, caffeinated tea, caffeinated soda, and chocolate), in the 72 h prior to dosing.

On day 7 (or earlier in the event of early termination), after collecting blood samples and completing standard safety assessments, participants were discharged from the clinic. On day 10, participants returned to the clinic for a follow-up safety evaluation. Participants were contacted by telephone 30 days post-dose for further safety follow-up.

### PET and MRI procedures

PET and MRI procedures were carried out at the Johns Hopkins Hospital. PET was obtained with a high-resolution research tomography (HRRT) scanner [17, 18], with 207 image slices of approximately 1.2 mm thickness in 2.4–2.8 mm transaxial spatial resolution. Three PET scans were performed per participant: at baseline (i.e., during the period from day − 29 to day − 1), at 4 h post-dose (day 1), and at 23.5 h post-dose (day 2). These timings were to reflect baseline, maximum plasma concentration (C_max_), and post-C_max_ time points, respectively. In each PET session, the participant had an indwelling intravenous (IV) catheter inserted, and was positioned on the scanner bed using a thermoplastic mask to reduce head motion. A transmission scan was performed using a ^137^Cs source to correct for the effects of attenuation. Dynamic PET started with a bolus IV injection of the selective dopamine D_2_/D_3_ receptor antagonist, [^11^C]raclopride, and lasted for 90 min. The PET radiopharmaceutical, [^11^C]raclopride, was produced in the Johns Hopkins radiochemistry facility in high specific activity using methods previously published [19]. The dynamic PET scans were reconstructed into 30 frames (four 15-s, four 30-s, three 1-min, two 2-min, five 4-min, and twelve 5-min frames), correcting for attenuation, scatter, and dead time. Each PET frame consisted of 256 by 256 by 207 (axial) voxels (1.2 mm cubic), decay corrected to the tracer injection time.

Structural spoiled gradient recalled (SPGR) MRI was performed at screening, using a 3.0 T SIGNA scanner (GE Healthcare, Chicago, IL, USA), to rule out cerebral lesions, to define volumes of interest and to perform PET-to-MRI coregistration and spatial normalization.

### PET analysis

Volumes of interest (VOIs) were defined on individual participant MRI images for the putamen, caudate nucleus, and cerebellum gray matter, and transferred to the PET space using PET-to-MRI coregistration parameters from SPM5’s coregistration module [20]. Then, VOIs were applied to PET frames to generate time–activity curves. The primary outcome variable, binding potential relative to non-displaceable binding (BP_ND_) [21], was determined by the multilinear reference method with two parameters (MRTM2) [22], with the cerebellum as the reference region. Estimates of dopamine D_2_/D_3_ receptor occupancy by brexpiprazole were obtained as follows: occupancy (%) = (BP_ND_^B^ − BP_ND_^D^) / BP_ND_^B^ × 100, where superscripts B and D indicate BP_ND_ at baseline and post-dose scans, respectively. Plots of occupancy (pooled over participants separately for the putamen and caudate nucleus) versus brexpiprazole plasma concentration (C_p_) were fitted by the following saturation equation: occupancy = O_max_ × C_p_ / (C_p_ + EC_50_), where O_max_ is the maximum obtainable D_2_/D_3_ receptor occupancy, and EC_50_ is the plasma concentration predicted to provide 50% of O_max_. In the fitting procedures, O_max_ was 1) fixed at the theoretical maximum (100%), and 2) estimated in addition to EC_50_; the O_max_ associated with a lower Akaike information criterion [23] was taken as optimal. Functional maps of BP_ND_ were generated by voxel-wise MRTM2 to visually confirm dose-dependent changes of BP_ND_ from the baseline to post-dose scans. For this purpose, functional maps were spatially normalized to a standard brain by applying parameters of PET-to-MRI coregistration and spatial normalization of MRI in one step [24], and smoothed by a Gaussian kernel of 8 mm full-width at half-maximum to compensate for insufficiency of spatial normalization.

### Pharmacokinetic procedures and analysis

Full details of the pharmacokinetic procedures and analysis are presented in the Online Resource. In summary, the plasma concentration of brexpiprazole and its main metabolite, DM-3411, were determined using high-performance liquid chromatography with tandem mass spectrometric detection (HPLC-MS/MS). The following pharmacokinetic parameters were determined: C_max_, time to C_max_ (t_max_), area under the concentration–time curve to the last observable concentration at time t (AUC_t_) and to infinity (AUC_∞_), terminal-phase elimination half-life (t_½,z_), and apparent clearance of drug from plasma after extravascular administration (CL/F).

### Safety assessments and analysis

The safety of brexpiprazole was evaluated at both centers by the reporting of adverse events (AEs) and by standard safety assessments at various time points, including physical examination, electrocardiograms, vital signs, and clinical laboratory tests. All participants who took a dose of brexpiprazole were included in the safety analysis.

## Results

### Disposition and demographics

A total of 15 healthy volunteer participants were enrolled, all of whom took a single dose of oral brexpiprazole (study flow shown in Fig. S3 in the Online Resource). Two participants each received brexpiprazole 0.25 mg, 0.5 mg, 1 mg, 2 mg, 4 mg, and 6 mg; three participants received brexpiprazole 5 mg. Fourteen participants (93.3%) completed the trial; one participant (5 mg dose) was withdrawn by the investigator due to problems with the PET scanner.

In the enrolled sample, the baseline mean (standard deviation) age was 33.9 (6.8) years, weight was 81.7 (13.4) kg, and BMI was 26.6 (4.2) kg/m^2^; 93.3% of participants (14/15) were male, 86.7% (13/15) were Black/African American, and 13.3% (2/15) were Asian.

### Dopamine D_2_/D_3_ receptor occupancy

**Fig. 1:**
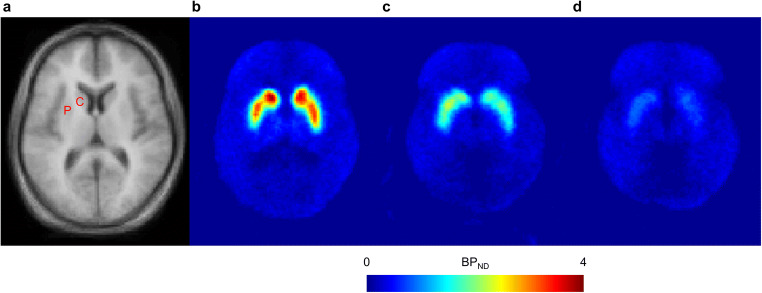
Mean trans-axial **a** MRI (*n* = 13) and **b**–**d** PET BP_ND_ images with [^11^C]raclopride, at a level showing the putamen and caudate nucleus. Panel **b** shows mean BP_ND_ at baseline (*n* = 13). Panel **c** shows mean BP_ND_ for arbitrarily selected ‘low’ brexpiprazole plasma concentration (< 20 ng/mL; *n* = 13). Panel **d** shows mean BP_ND_ for arbitrarily selected ‘high’ brexpiprazole plasma concentration (> 20 ng/mL; *n* = 12). *BP*_*ND*_ binding potential relative to non-displaceable binding, *C* caudate nucleus, *MRI* magnetic resonance imaging, *P* putamen, *PET* positron emission tomography

**Fig. 2:**
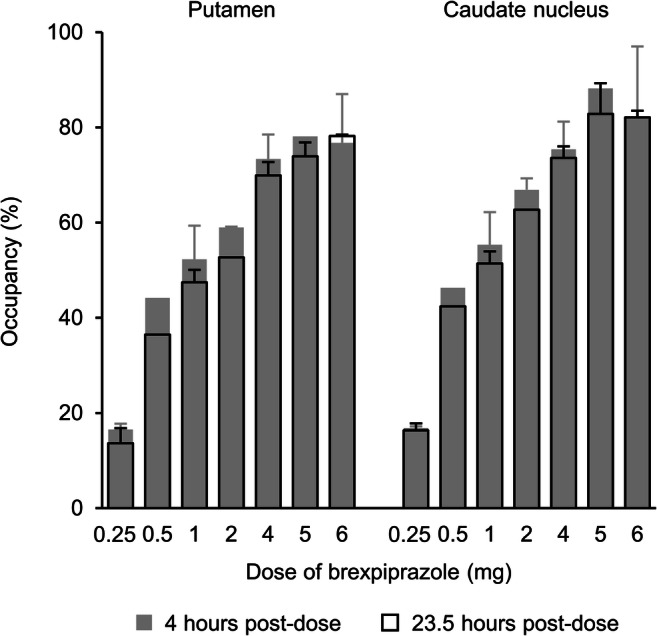
Mean dopamine D_2_/D_3_ receptor occupancy at 4 h and 23.5 h following a single oral dose of brexpiprazole (*n* = 2 per dose group except where there is no error bar, *n* = 1), as a function of dose. Error bars represent standard deviation

Trans-axial BP_ND_ images at a level showing the putamen and caudate nucleus are shown in Fig. 1, at baseline and for low and high brexpiprazole plasma concentrations. At 4 h post-dose, the mean D_2_/D_3_ receptor occupancy in the putamen and caudate nucleus increased with brexpiprazole dose (from < 20% with brexpiprazole 0.25 mg), leveling out at 77–88% with brexpiprazole 5 mg and 6 mg (Fig. 2). A similar pattern of D_2_/D_3_ receptor occupancy was observed at 23.5 h post-dose (Fig. 2), leveling out at 74–83% with brexpiprazole 5 mg and 6 mg. Single doses of 2–4 mg resulted in D_2_/D_3_ receptor occupancies of 59–75% at 4 h and 53–74% at 23.5 h post-dose.

**Fig. 3:**
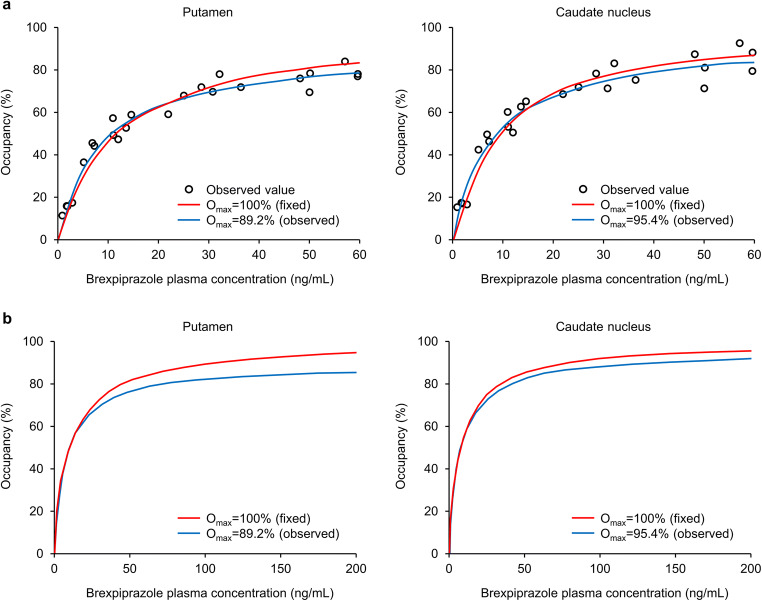
Dopamine D_2_/D_3_ receptor occupancy versus brexpiprazole plasma concentration **a** following a single oral dose (*n* = 12), and **b** extrapolated to expected multiple-dose concentrations. The mean plasma concentration across four time points spanning the duration of PET data acquisition was used for each data point. O_max_ maximum obtainable receptor occupancy, *PET* positron emission tomography

Estimates of O_max_ and EC_50_ and model prediction curves were similar at 4 and 23.5 h post-dose, and so the datasets were combined. Plots of D_2_/D_3_ receptor occupancy as a function of brexpiprazole plasma concentration are shown in Fig. 3a. Plasma concentrations of 60 ng/mL corresponded to approximately 80–90% D_2_/D_3_ receptor occupancy in the putamen and caudate nucleus. Based on the combined data, estimates of O_max_ and EC_50_ were 89.2% and 8.13 ng/mL, respectively, in the putamen, and 95.4% and 7.75 ng/mL, respectively, in the caudate nucleus. Fixing O_max_ at 100%, estimates of EC_50_ were 11.5 ng/mL in the putamen and 8.99 ng/mL in the caudate nucleus. Akaike information criterion values were lower for (and thus supported using) estimated O_max_ in the putamen, and fixed O_max_ (100%) in the caudate nucleus.

Extrapolations of the predicted D_2_/D_3_ receptor occupancy beyond a brexpiprazole plasma concentration of 60 ng/mL based on estimates of O_max_ and EC_50_ are shown in Fig. 3b.

### Pharmacokinetic profile

**Fig. 4:**
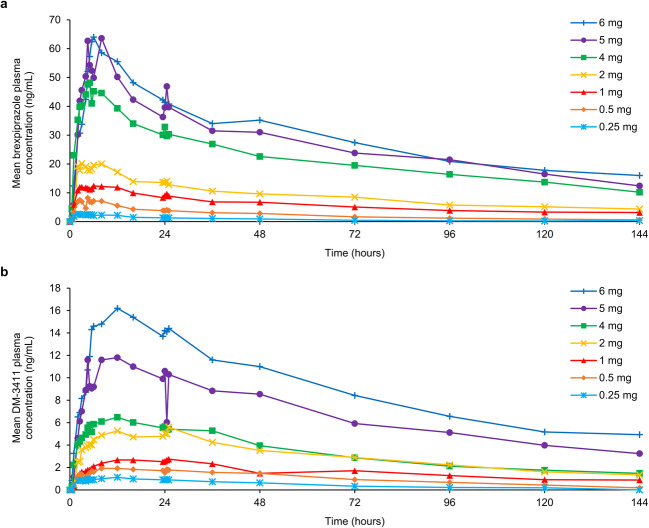
Mean **a** brexpiprazole and **b** its main metabolite, DM-3411, plasma concentration over time following a single oral dose of brexpiprazole (*n* = 2 per dose group)

Mean brexpiprazole and DM-3411 plasma concentrations versus time following administration of a single, oral dose of brexpiprazole are shown in Fig. 4. A summary of the pharmacokinetic parameters of brexpiprazole and DM-3411 are shown in Table 1. Brexpiprazole AUC_∞_ and C_max_ increased approximately proportional to dose.

**Table 1:** Mean pharmacokinetic parameters of brexpiprazole and its main metabolite, DM-3411, following a single oral dose of brexpiprazole

Dose	0.25 mg (*n* = 2)	0.5 mg (*n* = 2)	1 mg (*n* = 2)	2 mg (*n* = 2)	4 mg (*n* = 2)	5 mg (*n* = 2)	6 mg (*n* = 2)
Brexpiprazole							
C_max_ (ng/mL)	2.60	7.68	13.1	20.9	50.5	68.7	64.1
t_max_ (h)	2.24	4.34	7.48	4.96	5.71	6.54	9.34
AUC_t_ (ng.h/mL)	106	332	838	1280	3100	3870	4220
AUC_∞_ (ng.h/mL)	134	381	924^a^	2300	4170	5030	7040
t_½,z_ (h)	47.9	55.6	124^a^	132	73.4	74.6	95.1
CL/F (mL/h/kg)	25.7	21.6	14.2^a^	12.2	11.3	15.6	14.2
DM-3411							
C_max_ (ng/mL)	1.13	2.06	2.84	5.66	6.78	12.2	17.4
t_max_ (h)	12.0	20.2	20.5	25.7	11.2	24.0	11.4
AUC_t_ (ng.h/mL)	61.0	144	230	430	479	955	1290
AUC_∞_ (ng.h/mL)	84.8	170	326	724^a^	724	1470^a^	ND
t_½,z_ (h)	42.7	47.6	77.8	50.9^a^	120	68.4^a^	ND

^a^*n* = 1 (data missing for 1 participant)

*AUC*_*t/∞*_ area under the concentration–time curve to the last observable concentration at time t/to infinity, *CL/F* apparent clearance of drug from plasma after extravascular administration, *C*_*max*_ maximum plasma concentration, *ND* not determined (data missing for both participants), *t*_*max*_ time to C_max_, *t*_*½,z*_ terminal-phase elimination half-life

### Safety

Overall, 11/15 participants (73.3%) reported at least one treatment-emergent adverse event (TEAE). The most commonly reported TEAEs were postural orthostatic tachycardia syndrome (4 participants, 26.7%), nausea (3 participants, 20.0%), and headache (3 participants, 20.0%). A list of TEAEs occurring in ≥ 2 participants by dose is presented in Table S1 (Online Resource). The majority of TEAEs occurred at the higher brexpiprazole doses (4 mg, 5 mg, and 6 mg). All TEAEs were mild or moderate in severity. There were no discontinuations from the study due to TEAEs or due to electrocardiogram, vital sign, or clinical laboratory abnormalities.

## Discussion

In this open-label study in healthy participants, single-dose administration of oral brexpiprazole in the dose range of 0.25–6 mg resulted in dose-dependent increases in striatal D_2_/D_3_ receptor occupancy, and dose-dependent increases in C_max_ and AUC_∞_. This result aligns with a Japanese pharmacokinetics study in patients with schizophrenia, in which multiple-dose administration of oral brexpiprazole (1, 4, and 6 mg/day) increased C_max_ and AUC in a dose-dependent manner; steady state, based on mean brexpiprazole plasma concentrations in pre-dosing samples, was estimated to be reached after 10 days [25]. Furthermore, a recent PET study by Girgis et al. in the United States demonstrated robust dose-dependent occupancy at D_2_ receptors in 12 patients with schizophrenia following 10 days’ administration of brexpiprazole (1 or 4 mg/day) [26].

The recommended dose range for brexpiprazole in schizophrenia is 2–4 mg/day [27]. In the present study, single doses of 2–4 mg resulted in D_2_/D_3_ receptor occupancies of 59–75% at 4 h in the putamen and caudate nucleus, and 53–74% at 23.5 h. By extrapolating the observed single-dose data, multiple doses of 2 mg/day and above are expected to result in D_2_/D_3_ receptor occupancies of above 80%. The Girgis et al. study found a slightly lower steady-state D_2_ receptor occupancy (62–80% with the 4 mg dose), attributed to the choice of radioligand: [^11^C]-(+)-PHNO, a D_3_-preferring D_2_/D_3_ receptor agonist, rather than [^11^C]raclopride, a D_2_-preferring D_2_/D_3_ receptor antagonist (K_d_ = 1.43 nM for D_2A_ receptors and 1.58 nM for D_3_ receptors) [26, 28]. Whereas the brexpiprazole 2 mg and 4 mg doses have similar efficacy on schizophrenia symptoms when averaged across clinical trial samples [29], individual patients may benefit from different doses, and this individual response will be influenced by receptor occupancy.

In the present study, estimates of O_max_ and EC_50_ were 89.2% and 8.13 ng/mL, respectively, for the putamen, and 95.4% and 7.75 ng/mL for the caudate nucleus. These values are similar to those of aripiprazole in patients with schizophrenia, as observed using the D_2_/D_3_ receptor antagonist, [^18^F]fallypride: 92% and 10 ng/mL for the putamen, and 92% and 9 ng/mL for the caudate nucleus [30].

Brexpiprazole’s D_2_/D_3_ receptor occupancy was comparable at 4 h and at 23.5 h post-dose (of note, the half-life of brexpiprazole is approximately 91 h [27], and the half-life of aripiprazole is approximately 75 h [31]). This stable occupancy over 24 h supports the observed efficacy of once-daily dosing of brexpiprazole in schizophrenia and MDD [2–8]. The time to maximum plasma concentration for brexpiprazole 2–4 mg was 5–6 h in this study, and is reported as within 4 h in the prescribing information [27], comparable to that of oral aripiprazole tablets (3–5 h) [31].

A single dose of brexpiprazole was generally safe and well tolerated at levels up to approximately 90% striatal D_2_/D_3_ receptor occupancy in this sample of healthy participants. Overall in the schizophrenia clinical development program, brexpiprazole 2–4 mg/day was well tolerated, and no TEAEs had an incidence of ≥ 5% and twice that of placebo in short-term clinical efficacy studies [32]. Similarly, adjunctive brexpiprazole 1–3 mg/day was well tolerated in the MDD clinical development program, with only increased weight and akathisia occurring with an incidence of ≥ 5% and twice that of placebo in short-term clinical efficacy studies; these AEs generally did not lead to discontinuation [33]. The recommended dose of adjunctive brexpiprazole in MDD (2–3 mg/day) factors in CYP considerations; concomitant use of brexpiprazole with strong CYP2D6 inhibitors (e.g., paroxetine, fluoxetine) increases the exposure of brexpiprazole compared to the use of brexpiprazole alone [27].

There are two major contributions of this study to the academic understanding of the efficacy of dopamine partial agonists. Firstly, the study supports and further confirms the rationale for targeting > 80% occupancy of D_2_/D_3_ receptors by dopamine partial agonists for clinical effect. Indeed, in a previous collaboration, Johns Hopkins University and Otsuka carried out the first human D_2_/D_3_ receptor target engagement study with the partial agonist, aripiprazole [13]. Using the same PET radiotracer as the present study ([^11^C]raclopride), thereby allowing direct comparison between studies, aripiprazole had a steady-state occupancy of up to 95% at efficacious doses, with no increase in extrapyramidal symptoms [13]. (Of note, compared with aripiprazole, brexpiprazole has lower intrinsic activity at D_2_/D_3_ receptors, meaning that its intrinsic activity falls between that of aripiprazole and those of ‘pure’ antagonists [1].) In contrast, ‘pure’ D_2_ receptor antagonists are associated with dose-limiting side effects (such as extrapyramidal symptoms) above the therapeutic window of 65–80% occupancy [9–12]. Thus, the present results suggest that >80% occupancy of D_2_/D_3_ receptors is a ‘benchmark’ for dopamine partial agonists for the treatment of schizophrenia. The results of this single-dose, Phase 1, translational study in healthy volunteers were used to determine target dose ranges for subsequent registrational efficacy and safety studies of brexpiprazole in schizophrenia.

Secondly, since D_3_ receptor availability was not appreciably reduced by brexpiprazole treatment in the Girgis et al. study (as measured using PHNO) [26], the efficacious doses indicated by the present results may correspond to D_2_ receptor occupancy, rather than D_3_ receptor occupancy. Furthermore, given the known greater sensitivity to endogenous dopamine of agonist PET ligands (i.e., PHNO) versus antagonist PET ligands (i.e., raclopride) [34, 35], brexpiprazole’s lower occupancy with PHNO could allude to greater endogenous dopamine as a result of the partial dopamine agonism. Hence, by direct comparison of the raclopride results with the PHNO results, it can be hypothesized that endogenous dopamine may be a feature of brexpiprazole at D_2_ receptor sites. This may be a more straightforward explanation of the lower occupancy in the Girgis et al. study, as opposed to the rather complex mechanism of G protein affinity changes described by the authors [26].

The conclusions of this PET study are limited by its small sample size (*N* = 15), and the chance finding of a predominantly male, Black/African American sample (similarly, the Girgis et al. study had a small [*N* = 12] and predominantly Black/African American sample [26]). Nonetheless, the data were sufficient to fully describe brexpiprazole’s D_2_/D_3_ receptor dose–occupancy curve, and, in combination with pharmacokinetic outcomes, were sufficient to reliably select doses for registrational efficacy and safety studies in schizophrenia and MDD. With regard to dosing, based on prior tolerability data, the highest dose in the present study was 6 mg, limiting observations up to approximately 90% receptor occupancy. Finally, the study used single dosing rather than multiple dosing, which does not reflect use in clinical practice, although modeling was used to predict occupancies after multiple dosing.

## Conclusions

This was the first PET imaging study with brexpiprazole, and the second PET imaging study (after a study with aripiprazole) of target engagement by a dopamine partial agonist in healthy participants. In conclusion, the occupancy of dopamine D_2_/D_3_ receptors by brexpiprazole correlated with dose in healthy participants, and was consistent with the approved, clinically active, doses of 2–4 mg/day for schizophrenia and 2–3 mg/day for the adjunctive treatment of MDD [27]. The results of this study guided the dose selection in subsequent clinical trials, which ultimately resulted in the approval of brexpiprazole for the treatment of schizophrenia and the adjunctive treatment of MDD. These data, together with the earlier PET findings with aripiprazole, highlight the translational value of D_2_/D_3_ receptor occupancy assessment in the development of future dopamine modulators for the treatment of patients with schizophrenia.

## Electronic supplementary material

ESM 1 (DOCX 880 kb).

## Data Availability

To submit inquiries related to Otsuka Clinical Research, or to request access to individual participant data (IPD) associated with any Otsuka clinical trial, please visit https://clinical-trials.otsuka.com/. For all approved IPD access requests, Otsuka will share anonymized IPD on a remotely accessible data sharing platform.
