# Correction to: An open-label, positron emission tomography study of the striatal D_2_/D_3_ receptor occupancy and pharmacokinetics of single-dose oral brexpiprazole in healthy participants

**DOI:** 10.1007/s00228-020-03071-z

**Published:** 2021-01-04

**Authors:** Dean F. Wong, Arash Raoufinia, Patricia Bricmont, James R. Brašić, Robert D. McQuade, Robert A. Forbes, Tetsuro Kikuchi, Hiroto Kuwabara

**Affiliations:** 1 Present Address: Lab of CNS Neuropsychopharmacology And Multimodal Imaging (CNAMI), Mallinckrodt Institute of Radiology, Washington University in St. Louis, 4525 Scott Avenue Suite 3114, St. Louis, MO 63110 USA; 2 Otsuka Pharmaceutical Development & Commercialization Inc., Princeton, NJ USA; 3 Section of High Resolution Brain PET, Division of Nuclear Medicine and Molecular Imaging, The Russell H. Morgan Department of Radiology and Radiological Science, Johns Hopkins University School of Medicine, Baltimore, MD USA; 4 Otsuka Pharmaceutical Co., Ltd., Tokushima, Japan

**Correction to: European Journal of Clinical Pharmacology**

10.1007/s00228-020-03021-9

The correct affiliation 1 is presented in this paper.

The original article has been corrected.

