# Supplementary material for: An open-label, positron emission tomography study of the striatal D2/D3 receptor occupancy and pharmacokinetics of single-dose oral brexpiprazole in healthy participants
Source: Eur J Clin Pharmacol. 2020 Nov 16;77(5):717–25. doi: 10.1007/s00228-020-03021-9 (PMC8032567; doi:10.1007/s00228-020-03021-9)
Supplement: Supplementary file 1 — (DOCX 880 kb). [file 228_2020_3021_MOESM1_ESM.docx]

## Supplemental material

**An open-label, positron emission tomography study of the striatal D_2_/D_3_ receptor occupancy and pharmacokinetics of single-dose oral brexpiprazole in healthy participants**

*European Journal of Clinical Pharmacology*

Dean F. Wong, MD, PhD; Arash Raoufinia, PharmD; Patricia Bricmont, PhD; James R. Brašić, MD, MPH; Robert D. McQuade, PhD; Robert A. Forbes, PhD; Tetsuro Kikuchi, DVM, PhD; Hiroto Kuwabara, MD, PhD

Corresponding author: Dean F. Wong, MD, PhD; Johns Hopkins Radiology, Psychiatry, Neuroscience, Neurology, Environmental Health and Engineering, Carey Business School, Section of High Resolution Brain PET, Division of Nuclear Medicine and Molecular Imaging, Baltimore, MD, USA; e-mail: dfwong@wustl.edu and dean.f.wong@gmail.com

#### Fig. S1 Chemical structure of brexpiprazole


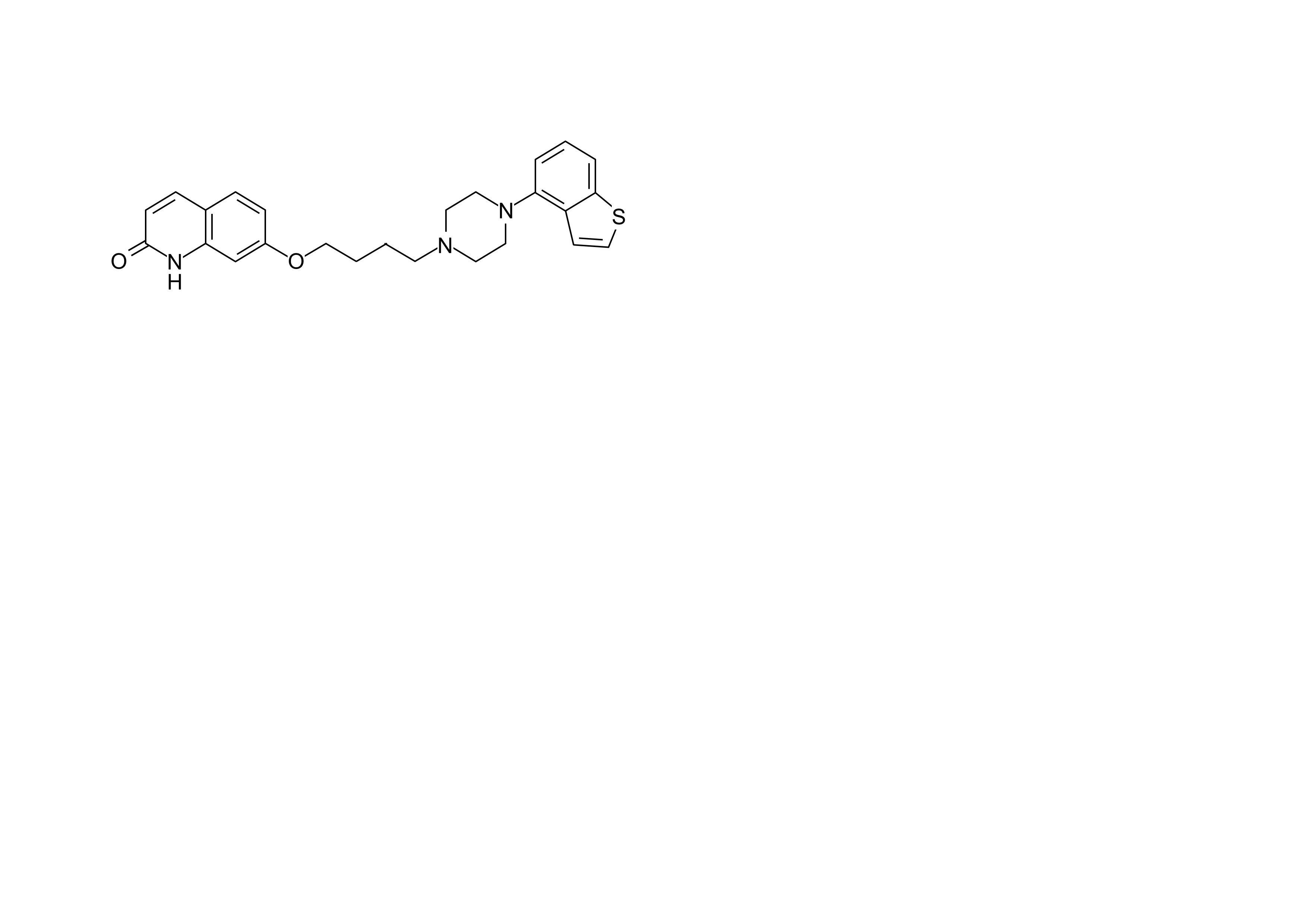


Source: Maeda K, Sugino H, Akazawa H et al (2014) Brexpiprazole I: *in vitro* and *in vivo* characterization of a novel serotonin–dopamine activity modulator. J Pharmacol Exp Ther 350:589–604

#### Fig. S2 Study design


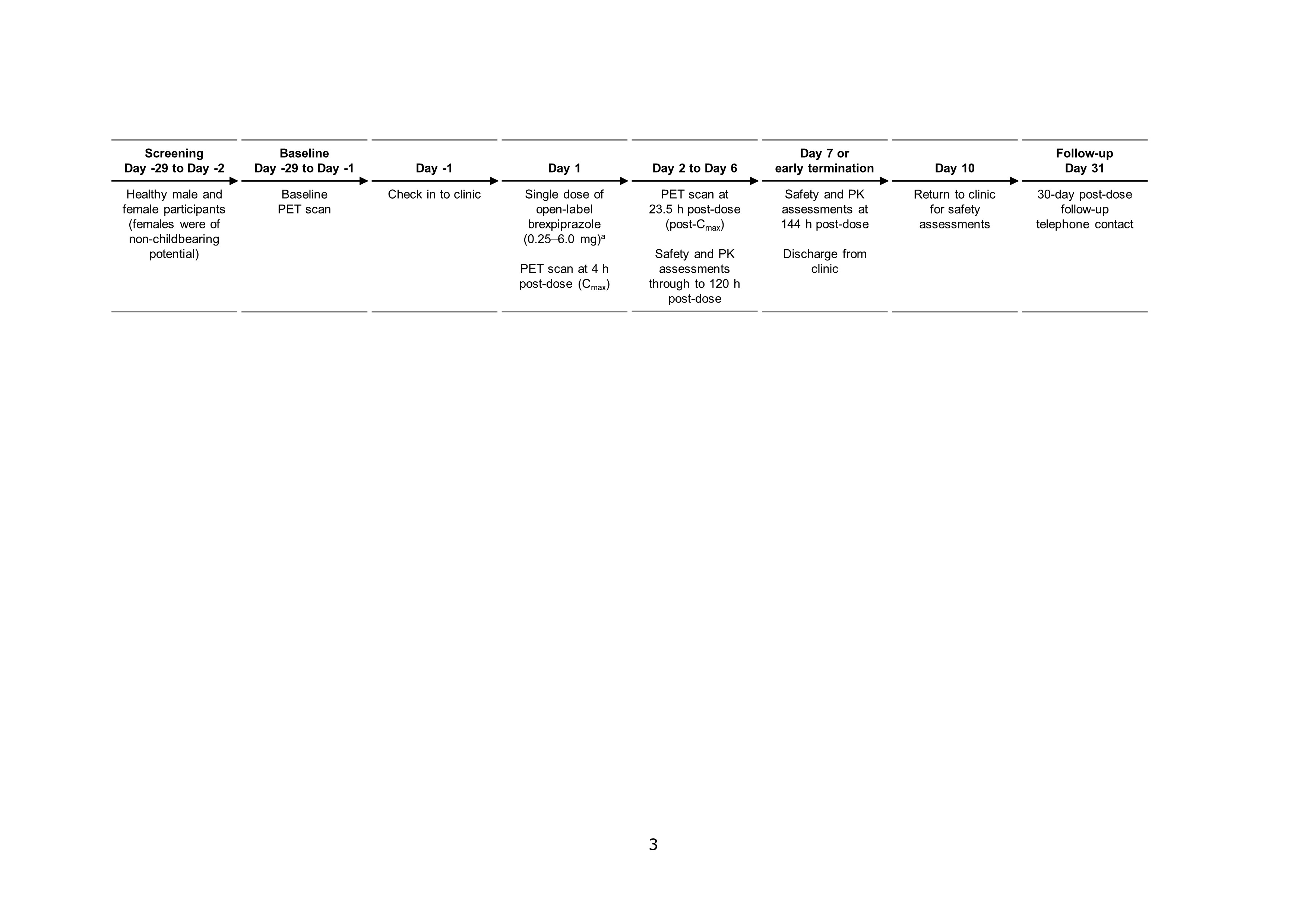


^a^The first 2 participants received 0.5 mg. The dose for each subsequent set of participants was determined based on results from the previous 2 participants: the dose could be increased, repeated, or decreased. An approximate 10-day lag between the dosing of each set of participants allowed review of prior results to determine the appropriate action.

*C_max_* maximum plasma concentration, *PET* positron emission tomography, *PK* pharmacokinetic.

#### Pharmacokinetic procedures

Blood samples were taken pre-dose and at approximately 0.5, 1, 2, 2.5, 3, 4, 4.5, 5, 5.5, 6, 8, 12, 16, 23.5, 24, 24.5, 25, 36, 48, 72, 96, 120, and 144 hours post-dose, or at the time of early termination. The plasma concentration of brexpiprazole and its main metabolite, DM-3411, were determined using high-performance liquid chromatography with tandem mass spectrometric detection (HPLC-MS/MS). Brexpiprazole and metabolites were extracted from human plasma samples by protein precipitation. After evaporation under nitrogen, the residue was reconstituted in acetonitrile:water (15:85). A 10−15 μL aliquot of this solution was then injected with a Shimadzu autoinjector (Kyoto, Japan). Chromatographic separation was achieved using a Waters XTerra RP18 (50 x 2.1 mm) (Milford, MA, United States), and analyzed using HPLC-MS/MS. The system suitability was verified by six injections of quality control (QC) samples containing all analytes. The method was linear over the range of 0.300 to 100 ng/mL for brexpiprazole and metabolites. For each batch of samples processed, the calculated concentrations of at least two thirds of the QC samples were within 15% of nominal. Over the course of the study, at each QC sample concentration, the relative standard deviation values were within 12.6% and the accuracy values were within ±11.1% for brexpiprazole and metabolites. Also, based on QC sample performance, there were no apparent stability problems encountered during the sample analysis. The incurred sample reassay test, in which a total of five samples from three participants were chosen for reassay, demonstrated adequate accuracy in that for at least two thirds of the samples the difference between the initial and incurred sample reanalysis results were within 20% of the mean of the two results.

#### Pharmacokinetic analysis

For calculation of descriptive statistics of concentration values, any values below the quantitation limit (BQL) were set to zero. For pharmacokinetic calculations, concentration values BQL prior to the first sample with measurable concentrations were set to zero. Concentration values BQL following the last sample with a measurable concentration were excluded from pharmacokinetic calculations. If all concentrations for a given analyte for a participant were BQL, then that participant was excluded from the analysis.

For each participant, brexpiprazole and DM-3411 concentration–time data were analyzed using a non-compartmental method [1]. Actual blood sample times were used for pharmacokinetic calculations. The following pharmacokinetic parameters were determined for brexpiprazole and DM-3411: maximum plasma concentration (C_max_), time to C_max_ (t_max_), area under the concentration–time curve to the last observable concentration at time t (AUC_t_) and to infinity (AUC_∞_), terminal-phase elimination half-life (t_½,z_), and apparent clearance of drug from plasma after extravascular administration (CL/F; for brexpiprazole only). Individual and summary tables of plasma concentrations by treatment (i.e., dose group) and time point were generated using descriptive statistics. Pharmacokinetic parameters were generated by treatment using descriptive statistics. Pharmacokinetic calculations were performed with WinNonlin Enterprise Version 5.2 (Pharsight Corp., St. Louis, MO, United States).

Reference

1. Jusko WJ (1992) Guidelines for collection and analysis of pharmacokinetic data. In: Applied pharmacokinetics: principles of therapeutic drug monitoring. 3rd edition. Eds. Evans WE, Schentag JJ, Jusko WJ. Vancouver, WA: Applied Therapeutics, Inc., pp 2-1–2-43

#### Fig. S3 Study flow


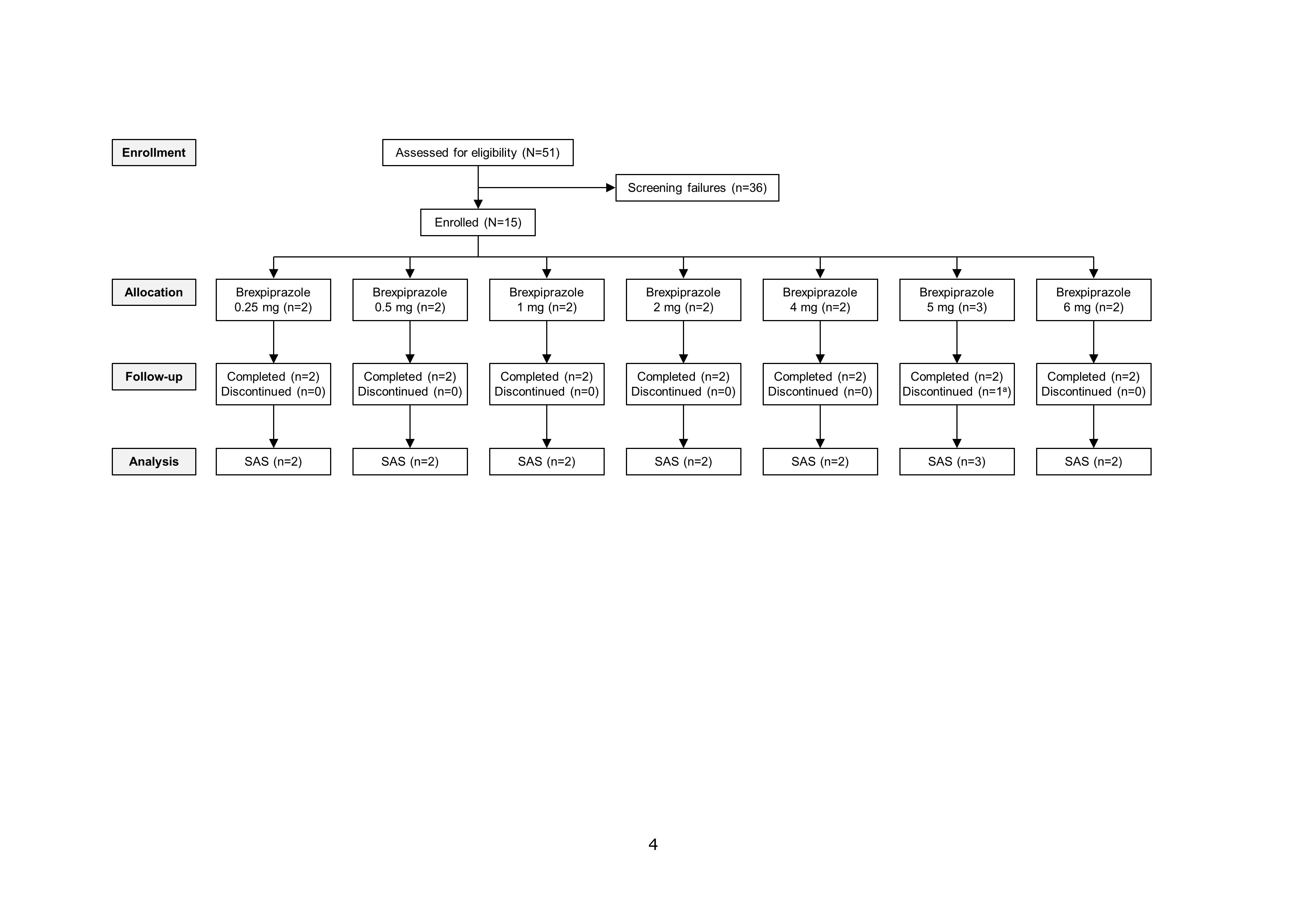


^a^Withdrawn by investigator due to problems with the PET scanner.

*PET* positron emission tomography, *SAS* safety analysis set.

#### Table S1 Treatment-emergent adverse events occurring in ≥2 participants overall following a single oral dose of brexpiprazole

| Dose | 0.25 mg (n=2) | 0.5 mg (n=2) | 1 mg (n=2) | 2 mg (n=2) | 4 mg (n=2) | 5 mg (n=3) | 6 mg (n=2) | Total (N=15) |
| --- | --- | --- | --- | --- | --- | --- | --- | --- |
| Postural orthostatic tachycardia syndrome | 1 | 1 | 0 | 0 | 0 | 1 | 1 | 4 (26.7) |
| Nausea | 0 | 0 | 0 | 0 | 1 | 1 | 1 | 3 (20.0) |
| Headache | 0 | 0 | 0 | 0 | 0 | 2 | 1 | 3 (20.0) |
| Tachycardia | 0 | 0 | 0 | 0 | 1 | 0 | 1 | 2 (13.3) |
| Fatigue | 0 | 0 | 0 | 0 | 0 | 1 | 1 | 2 (13.3) |
| Feeling hot | 0 | 0 | 0 | 0 | 0 | 1 | 1 | 2 (13.3) |
| Blood glucose increased | 0 | 0 | 0 | 0 | 1 | 1 | 0 | 2 (13.3) |
| Blood pressure increased | 0 | 0 | 0 | 0 | 1 | 1 | 0 | 2 (13.3) |
| Dizziness | 1 | 0 | 0 | 0 | 0 | 0 | 1 | 2 (13.3) |
| Somnolence | 0 | 0 | 0 | 0 | 1 | 1 | 0 | 2 (13.3) |
| Orthostatic hypotension | 0 | 0 | 0 | 0 | 1 | 1 | 0 | 2 (13.3) |

Values are n (%).
